# The European Society of Head and Neck Radiology Mentoring Programme: development and feedback during the first phase of the initiative

**DOI:** 10.1186/s13244-021-01119-x

**Published:** 2021-12-04

**Authors:** Steve Connor, Soraya Robinson

**Affiliations:** 1 School of Biomedical Engineering and Imaging Sciences, St Thomas’ Hospital, King’s College, London, UK; 2 Department of Neuroradiology, King’s College Hospital, Denmark Hill, London, UK; 3 Department of Radiology, Guy’s Hospital, 2nd Floor, Tower Wing, Great Maze Pond, London, UK; 4 Diagnose Zentrum Urania, Imagingurania, Laurenzerberg 2, 1010 Wien, Austria

**Keywords:** Mentoring, Education, Medical, Imaging, Diagnostic

## Abstract

There is increasing awareness of the benefits of formal mentorship programmes in radiology. In the context of the COVID 19 pandemic which impacted on education, professional engagement and networking within the wider radiological community, the European Society of Head and Neck Radiology (ESHNR) decided to develop a formal mentoring programme. The ESHNR mentoring initiative is novel in its scope, whereby European and international members of a subspecialty radiology society are matched into mentor–mentee pairings to disseminate good practice, knowledge and ideas. The purpose of this report is to describe the motivations, planning, challenges and early experience of the ESHNR mentoring programme together with initial feedback from the scheme.

The development of the programme and iterative modifications during the first phase of the scheme are described. The programme has enrolled 33 mentors and 27 mentees with international representation and 24 mentor–mentee pairs have participated in 2.6 (mean) meetings. The experience and benefits reported by the participating ESHNR members (mentees and mentors) were evaluated by a questionnaire at six months following the start of the programme. There were 80% of mentors and 88% of mentees who strongly agreed that the mentoring programme was rewarding rather than an obligation, and all participants reported that they would recommend the scheme to colleagues.

A formal mentoring programme has been established for an international subspecialty radiology society. The early experience is encouraging and suggests that it is both useful and sustainable. Our experiences may be of benefit to other subspecialty societies considering a mentoring programme.

## Key points

- There is increasing awareness of the benefits of formal mentoring in radiology.
- The development of an international subspecialty radiology society mentoring programme is described.
- Initial feedback found it was rewarding for the individuals and the society.

## Background

“Mentoring” is the action of advising or training another person, especially a less experienced colleague [1]. Whilst mentors may act as teacher, counsellor, coach and supervisor [2], the mentor can best be described as a trusted advisor and “guide” who helps the mentee develop and re-examine their own ideas, learning and development [3]. There is increasing awareness of the potential value of the mentoring partnership in medicine [4–8] with mentees gaining from professional support, knowledge, advice and career-specific skills, whilst mentors receive intellectual stimulation, learn new skills and are fulfilled by giving back to their institutions and specialty [9, 10]. Mentoring is perceived to be an important part of academic medicine [11] and is reported to influence professional and academic progress [6, 12]; however, the benefits of mentoring go far beyond the academic environment [13]. There are previous reports on its role and practice in radiology [9, 10, 14–24]. Studies have shown that mentoring of subspecialty fellows and early career radiologists may lead to greater research productivity and retention whilst also improved job satisfaction and patient care [17, 18]. However, formal programmes are not widely available [16, 17, 19] and the focus has been on academic radiology [17, 19, 21, 24].

The European Society of Head and Neck Radiology (ESHNR) was founded in January 1987 in order to advance knowledge, stimulate interest, develop methods and foster science in head and neck radiology. It performs a range of roles including teaching and research and encourages interaction between radiologists with an interest in this field. There are 686 members (as of 16/8/21) with widespread international representation from Europe and beyond. There is an executive committee of 14 fellows and administrative support is provided through the European Society of Radiology (ESR) office. In view of the society’s objectives and the cessation of normal functions during the COVID 19 pandemic, the concept of a ESHNR mentoring scheme for its members was proposed as an extension of its activities in 2020. The purpose of this report is to describe the motivations, planning, challenges, iterative adaptations and early experience of the mentoring programme, and to document some early outcomes and feedback. The report is structured to incorporate Standards for Quality Improvement Reporting Excellence in Education (SQUIRE-EDU) guidelines.

## Why?

In view of the accumulating evidence for the positive effects of mentoring in academic radiology and other settings [9, 10, 14–24], the ESHNR executive committee considered whether they should create their own formal mentoring programme. The initiative was in keeping with the aims of the society, which are to advance knowledge, to facilitate research and teaching, and to stimulate interest in head and neck radiology. Formal mentoring within an international subspecialty radiology society could offer an array of benefits for both the society and its individual members.

Firstly, the addition of a mentoring programme to the society’s range of functions and activities could raise the profile of the ESHNR, promote head and neck radiology, attract new members and expand, as well as rejuvenate the society. By directly engaging with the mentors and mentees, it was anticipated that there would be an improved sense of inclusivity, overcoming generational differences between members and facilitating both relationships and networking within the society. By enhancing the connection of members with the society, it hoped to create equal opportunities for them to contribute and become educational and research leaders within the ESHNR. The international spread of ESHNR members would enable the programme to disseminate good practice across the geographically diverse society and to help equalise opportunities and access. The common interest in head and neck radiology would also allow the mentors and mentees to focus on the particular challenges and issues most relevant to the subspecialty.

Secondly, the ESHNR was keen to help and enhance the careers of its younger members, to increase their opportunities and to give them confidence to progress as head and neck radiologists. Given the diversity of the society, this was felt to be most relevant where members had less access to any local mentoring infrastructure or career support. The executive committee of the ESHNR perceived there to be a wide variation in the availability of local mentoring resources for head and neck radiologists. In particular, it was recognised that early years consultants, who are practicing independently for the first time, may be overlooked. It was expected that their needs could be addressed by careful matching with a skilled and experienced mentor, who was familiar with the clinical, professional and personal challenges of the head and neck radiologist. In addition, the ability of the society to provide mentors from outside the mentee’s institution was felt to be advantageous, since it would avoid any conflicts of interest with regards to productivity and resources.

Finally, the ESHNR also recognised that the mentor–mentee programme should be a fulfilling two-way process and should enhance the professional life of the mentor as well. The concept of reverse mentoring is well established [25]. It was appreciated that the more experienced head and neck radiologist could learn much from their mentee and, in turn, the mentee would feel valued and consulted. By challenging the way their mentor thinks and by discussing topics such as new technology and innovations (e.g. social media and Artificial Intelligence) or evolving social perspectives, the mentee could help their mentor gain valuable insights and remain relevant.

The aspirations of the ESHNR mentoring programme were brought into stark focus by the consequences of the COVID 19 pandemic, whose radical impact on education and professional engagement is well recognised. Interaction with peers and networking through face-to-face ESHNR annual meetings or through European School of Radiology visiting fellowships was no longer possible, and the opportunities for developing short-term informal mentoring relationships were reduced. There was perceived to be a potential for a sense of isolation and career attrition amongst ESHNR members, which the society had an opportunity to address. In addition, all are familiar with the synchronous rapid development of online meetings and remote collaborations during this period [26]. The facility for web-based meetings between mentors and mentees across the world created an exciting new opportunity for international mentoring.

## Aims

It was hoped that the mentoring scheme would provide benefit to the individual mentors and mentees as well as the ESHNR society and would be enriched by the diversity of participants and matching. The operational objectives of the formal mentoring programme were to bring together ESHNR members in appropriate mentor–mentee matchings, to prepare them for the mentor–mentee relationship and to guide them through the one-year mentorship period. The structure and processes were expected to evolve on the basis of feedback, and it was anticipated the programme would grow. The ESHNR mentoring programme was not designed to replace but to complement any existing local mentoring strategies available. Of note, the mentoring process was not primarily intended to be a training tool, with the mentee-mentor pairings being encouraged to explore non-clinical areas (e.g., work-life balance, interactions with colleagues) in an open discussion that should include feedback from both parties. The key components to the programme were envisaged to be information on the intentions of the mentoring, administration of the programme, vetting of applicants, timely mentor–mentee matching, training resources, clear expectations and advice on conducting meetings, a defined duration of the programme and a process of feedback (Table 1).

**Table 1:** Key components of the mentoring programme

Information on the aims and motivations of the mentoring programme
Administrative process for the engagement and enrollment of members into the programme
Structure for the timely mentor–mentee matchings
Training resources for the mentors
Expectations and advice on conducting the mentor–mentee meetings
Defined duration of the programme and closure of the process
Process of feedback

## Planning the journey

The journey commenced in May 2020 with the idea being proposed to the ESHNR executive committee by the senior author (S.R.). The planning started in earnest in June 2020 with the formation of a core team (S.C./S.R.) and with administrative support from the ESHNR office. Over the following months, a series of mentee/mentor requirements, processes, documents and website content was developed. To be eligible for the mentoring programme, mentees were required to be ESHNR members who were in at least their fourth year of training or in their early years at a consultant level, whilst mentors were expected to have been in independent head and neck radiology practice for at least five years. Additional desirable qualities for the mentor included an interest in research and teaching as well as previous experience in mentoring, whilst prospective mentees were encouraged to question their specific needs prior to applying. Engagement in the programme was expected to be three years for mentors and one year for mentees (although they could reapply).

**Fig. 1:**
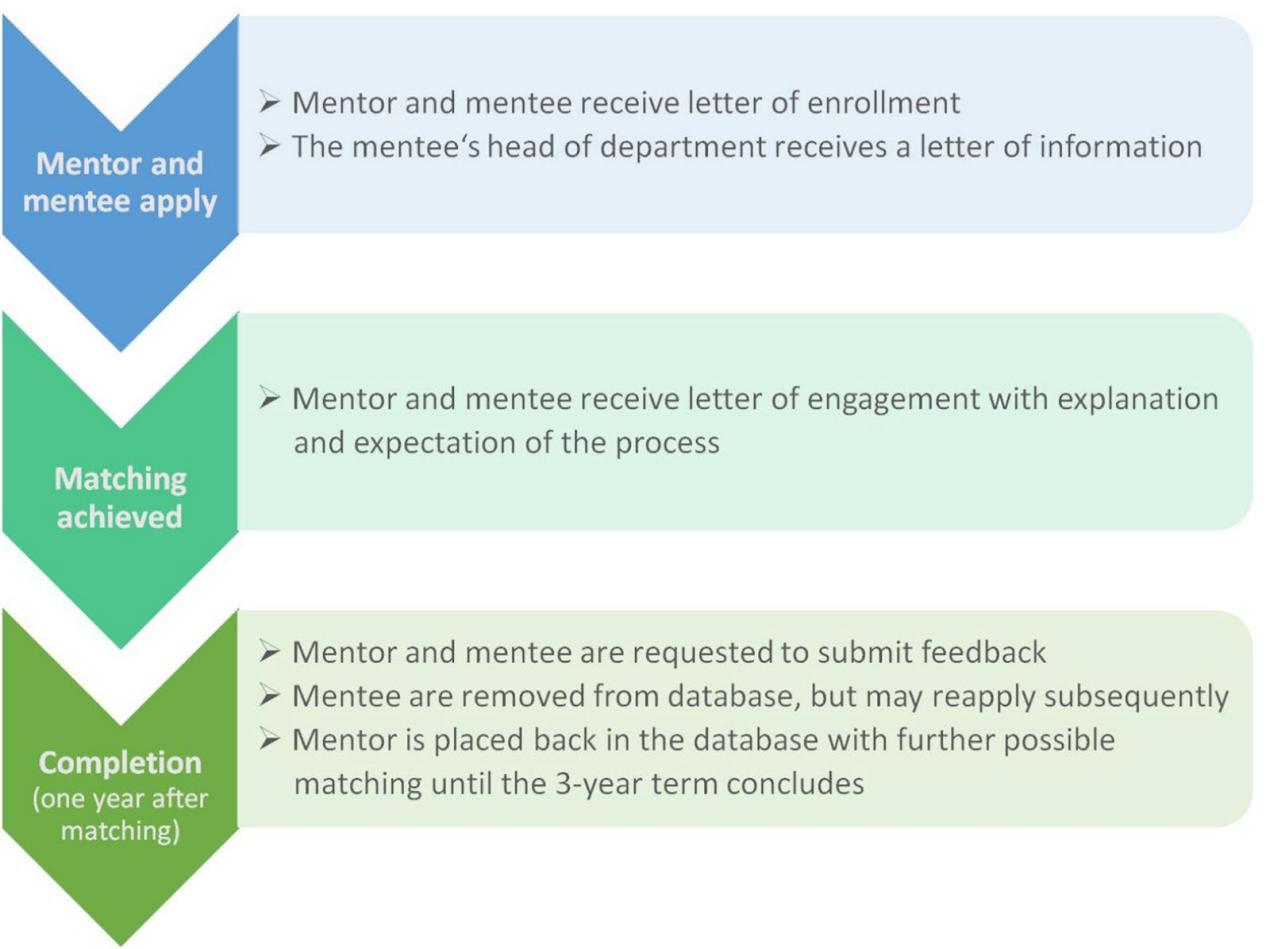
The key features of the mentoring process

The key features of the mentoring process (www.eshnr.eu/mentoring) (Fig. 1) comprised (1) application forms to be completed and submitted to the ESHNR (2) enrollment letters sent to mentors/mentees (3) letters sent to the head of training or head of department of prospective mentees (4) matching of mentor/mentees according to a priori criteria (Table 2) (5) engagement letters copied to both mentors and mentees to aid communication (6) recommendations on minimum frequency and number of mentoring meetings and (7) feedback forms and certificates sent to mentors and mentees on completion of the one year programme. All correspondence was to be by -mail and co-ordinated centrally by the ESHNR office. The applicants’ details were to be kept confidential on encrypted files by the ESHNR office with a database confirming dates of application and matching.

**Table 2:** Criteria for matching of mentors/mentees

*Order and process of matching*
Prioritised mentees deemed to lack opportunities for mentoring in head and neck radiology
By date of receipt of application
Performed centrally by consensus of the core team
*A priori criteria for choice of mentoring pairing*
Specific mentors/mentees could not be requested
Mentors/mentees should be from different countries (unless the same country requested due to language barriers)
Based on the specific needs and skills of the mentor and needs of the mentee on the application (e.g., academic mentor requested)

Specific documents and website content were formulated in order to support the process. Formal engagement letters and enrollment letters were supplemented by a series of website links to support self-directed mentoring training. A letter to the head of training/head of department explained the programme and how it should be seen as additional to any local mentoring process. Feedback forms were designed to determine whether the aims of the mentoring scheme had been achieved. Website content outlined the drivers and goals of the mentoring programme, the requirements of applicants, details of the matching process, expectations concerning the frequency and number of meetings, and advice on maintaining a successful mentor–mentee relationship.

As part of our “pre-launch checks” there were some specific concerns and decisions which warrant further explanation (Table 3).

**Table 3:** “Pre-launch checks”

Concern/issue	Decision/solution
Potential conflict with supervision and mentoring at local institution	Letter sent to the head of department/ trainer to explain the complementary role of the mentoring programme
Ensuring that procedures complied with General Data Protection Regulations (GDPR)	Legal advice sought through the ESR. Central collection of limited mentor and mentee personal information for the duration of their involvement in the programme was deemed as necessary for the functioning of this ESHNR activity and compliant with GDPR
Potential errors in transcription from scanned handwritten application forms	Use of interactive pdf with field entries for applications
Variable training duration and structure between countries complicating the definition of eligibility criteria for mentors and mentees	Use of inclusive and generalizable eligibility criteria
Potential for excessive administration in order to repopulate the mentor and mentee database on an annual basis	Decided that mentors would enrol on the programme for a 3-year duration
Requirement for timely matching process by consensus of the core team	Organised real time access to mentor/mentee spreadsheets on a shared drive which was updated and monitored by the office
Maximising the recruitment of mentors and mentees	Mentors were listed on the website to “showcase” the scheme. The mentoring programme was marketed by the ESHNR and announced at ESHNR webinar sessions
Lack of recognition for mentoring efforts	Certificates to be produced on completion

## The launch and early trajectory

The mentoring programme was launched in December 2020. Some initial challenges pertained to the imbalance in the recruitment between mentors and mentees, erroneous contact information, mentees disengaging from the programme and enquiries concerning the role and format of the mentoring process.

**Fig. 2:**
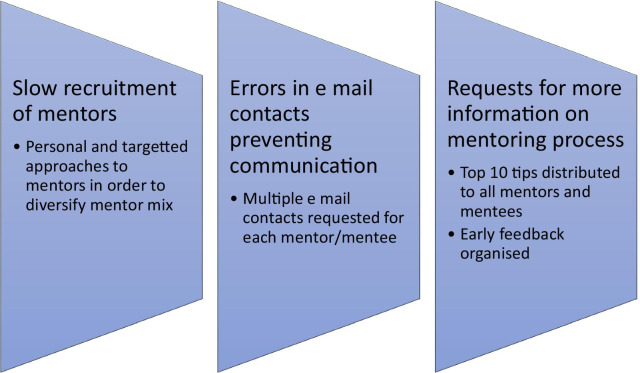
Iterative modifications and interventions

Firstly, initial recruitment of mentors did not keep pace with that of mentees. This was addressed by a round of personal approaches to prospective mentors, with emphasis on engaging from different countries in order to expand the geographic spread and those who were peri-retirement in order to vary the ages of the applicants. Secondly, human error in completing and transcribing the application forms prevented contact between several mentor/mentee pairings. It was subsequently decided that two different email contacts would be requested to aid communications. Finally, some mentees decided to discontinue the programme prior to any meetings due to changes in circumstance or misunderstanding the purpose of the mentoring process. In response to this and ongoing enquiries for information, the core team created and disseminated a “top ten tips for mentoring” to foster a successful mentoring relationship and to address some misconceptions (Table 4). An early feedback questionnaire (6 months into the mentoring programme) was also formulated to determine whether some key aims of the mentoring programme were being achieved. These iterative modifications and interventions are shown (Fig. 2).

**Table 4:** “Top ten tips” for mentoring

1	*Mentees should be pro-active and take the initiative* -Be prepared with topics you would like to discuss and even better… -Pre-warn your mentor about topics you would like to discuss
2	*Actively seek and be receptive to feedback from your mentor/mentee*
3	Mentors could come armed with 1–2 “pearls” or words of wisdom for each discussion-but **the process is far more purely than “education and knowledge”** -note there should be no expectation from the mentees that they should be offered fellowships and attachments by the mentors
4	Consider a **plan and set goals going forward** at the end of each session
5	**Be accessible**
6	Be **honest, open and respectful** of each other’s time
7	**Mutual effort and commitment** will energize the process
8	**Privacy and confidentiality** are absolute
9	It maybe that the mentor–mentee **relationship just does not work** (e.g., lack of “chemistry”) -this is no one’s fault -we hope you will persist for the opening 4 meetings at least
10	**This is a 2-way process**—mentors have as much to gain from the process as mentees

## Are we keeping on course? Outcomes of the scheme

Over the first six months, 33 mentors (22 male, 11 female) and 27 mentees (13 male, 14 female) enrolled in the ESHNR mentoring programme. The mentoring experience and motivations of the applicants were documented (Table 5). Mentors and mentees felt they could particularly contribute or benefit from research skills (e.g. grant application, writing papers, publications), educational advice (e.g. ESHNR diploma preparation, fellowship advice, courses to recommend), clinical work (e.g. how to initiate and improve multidisciplinary meetings and protocols), career development and planning (e.g. work-life balance, organisation and networking) and other skills and attributes (e.g. resilience, communication, assertiveness, leadership and organisation).

**Table 5:** Mentoring experience and motivations of the applicants

	Mentors (*n* = 33)	Mentees (*n* = 27)
*Prior experience of mentoring*	17	5
*Areas where applicants considered they could most contribute or benefit*		
Research	22	18
Education	23	23
Clinical	26	26
Career development	24	20
Other skills	15	15
*Most frequently stated motivations to join mentoring programme (in order of frequency)*	-Pass on and share experience/knowledge -Enjoy teaching -To help, motivate and support -Benefited from mentoring and would like to “give back” -Interacting with younger radiologists -Would benefit and learn from mentee	-Help with interpreting head and neck imaging -Developing new protocols and techniques -ESHNR diploma preparation -Developing services and multidisciplinary meetings -Academic /research skills -Career development -Hoping it would lead to a fellowship/observership

There were 24 mentors from Europe (including United Kingdom (*n* = 6), Austria (*n* = 4), Germany (*n* = 4) and Italy (*n* = 3)) and nine from outside Europe (including Brazil, USA, Singapore, India and Australia). The mentors had a mean of 17.5 years’ experience (range 5–34) in head and neck radiology. There were 19 mentees from Europe (including United Kingdom (*n* = 6), and Italy (*n* = 4)), and eight from outside Europe (including Brazil, Argentina, Mexico, Pakistan, Malaysia, Egypt, Nigeria and Australia). There were 23 mentees who were early years consultants and four mentees who were trainees. There were 27 mentor and mentee pairings initially matched. The mean interval from mentee application to mentoring engagement letters being sent was 57.6 days (range 51–68) for the first 14 pairs and 9.2 days (range 0–34) for the next 13 pairs. At six months following the start of the programme, one mentee had withdrawn from the program.

**Fig. 3:**
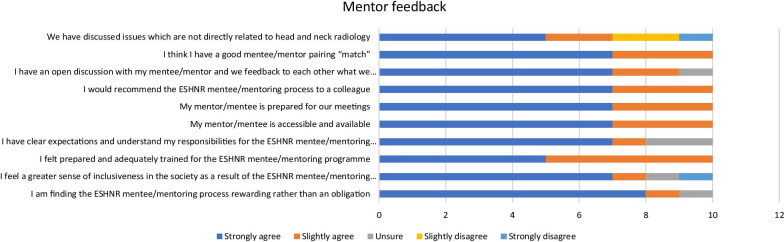

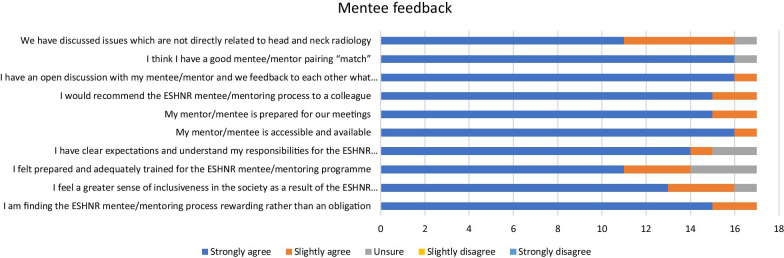
Responses from the 6-month feedback questionnaire

A six-month questionnaire was sent to the mentors and mentees on two occasions, which was administered centrally by the office. This gathered information on the number and nature of mentoring discussions, but primarily consisted of a series of questions designed to evaluate the aims of the mentoring program and the success of its processes so far. There were 32/54 respondents to the questionnaire. The mentors and mentees opinions and perceptions were rated on a Likert scale with additional free text answers requested (see supplementary material). Five respondents were yet to commence mentoring meetings at 6 months into the programme of which two had been unable to contact their mentor/mentee. Hence there were 27 (10 mentors and 17 mentees) respondents who were actively involved in mentoring meetings and who completed the questionnaire. These were anonymised for analysis and the results are shown in Fig. 3 for both mentors and mentees.

The mean number of mentoring meetings taking place in the first six months of the programme was 2.6 (range 1–6). Although there was some emphasis on specific head and neck radiology topics, protocols and techniques (e.g., head and neck cancer, salivary gland imaging, molecular imaging), a number of more general issues were reported to have been discussed (e.g. work-life balance, diploma preparation, educational resources, dealing with difficult colleagues, how to teach, academic systems, variations in healthcare organisations, radiology departmental workflow).

As an example of the range of mentoring discussions, one of the authors documented the mentoring discussion during the initial four mentoring meetings (Table 6). Specific approaches to the meetings reported by other mentors and mentees during the feedback included the use of powerpoint presentations, quizzes, exchanging interesting cases, discussing how they would adapt their practice as a result of the discussion and alternating the choice of subject matter.

**Table 6:** Sample mentoring meeting discussion points

	Meeting 1 (45 min)	Meeting 2 (30 min)	Meeting 3 (40 min)	Meeting 4 (40 min)
Mentor or Mentee proposed topic	Introductory meeting: led by mentor	Topic proposed by mentee	Topic proposed by mentee	Topic proposed by mentor
Main theme	Discussed our backgrounds and aspirations. Explained the mentoring role	How to deal with difficult colleague relationships and discussed developing multi-disciplinary meetings	Artificial intelligence in head and neck radiology	Temporal bone imaging-most important things I have learnt. Talked about webinar resources

The questionnaire feedback was generally positive from both groups, particularly the mentees (Fig. 3). The majority of respondents strongly agreed with the key aim that the mentoring programme was rewarding rather than an obligation (80% of mentors and 88% of mentees). There was approval of the structures and processes, with all mentors and mentees being accessible and prepared for meetings, and 96% of the participants felt that they had a good “match”. Moreover, all participants reported that they would recommend the scheme to colleagues, with 70% of mentors and 88% of mentees strongly agreeing with this statement. However, only 50% of mentors and 55% of mentees “strongly agreed” that they were prepared and adequately trained, and 15% of all participants were unsure of their expectations and responsibilities. Finally, only 59% of all participants had discussed issues not related to head and neck radiology and this was also evident in the free text reporting of the issues discussed in the mentoring meetings.

## Onwards and upwards: reflections on the journey so far and challenges ahead

With respect to whether the mentoring programme is meeting its aims for the individuals and the society, the 6-month feedback (Fig. 3) indicated that mentors and mentees found it rewarding, and that it generally resulted in greater inclusiveness within the ESHNR. Those enrolled in the programme demonstrated diversity in their international representation and their range of clinical experience. As for the mentoring process and structure, the matching was universally reported to be “good” or “very good” in the questionnaire responses and, despite some initial delays, it was subsequently performed in a timely manner and according to a priori criteria. The availability and accessibility of participants aided the organisation of meetings. The meetings appeared adequately prepared and well managed with open discussion and feedback. All participants volunteered that they would recommend the initiative, which is a positive indicator for the future growth of the programme.

However, improvements could be made in terms of preparing mentors and mentees and ensuring that their responsibilities are clearly established. This may also be reflected in the paucity of discussion about topics other than head and neck radiology, which may be addressed by education on the importance of these elements in the mentoring process. Although website material was made available and e-mail bulletins (e.g., “top 10 tips”) were employed, there may be a need for more resources (e.g., podcasts, manuals) or active interventions such as mentor online meetings or mentoring sessions and workshops at ESHNR meetings. However, soft mentoring skills are a feature of medical practice in most settings, and it is felt that the society should not be responsible for formal mentoring training.

A mixed qualitative and quantitative approach is usually employed to evaluate mentoring programmes [6], and our application of Likert scales and open-ended questions is in line with most previous reports. The Kirkpatrick model [6, 27] has been recommended to measure effectiveness of mentoring programmes with four sequential levels being described. However, this includes the evaluation of tangible outcomes such as publications and exam success, which is beyond the remit of the ESHNR mentoring programme. It should be noted that our questionnaire was not validated or piloted before use. It should also be appreciated that there was potential bias in our evaluation since the feedback was only received in 27 (46%) of those actively engaged in the programme, and it may have been selective towards those more motivated. In some cases, it was based on only limited contact (with only one meeting having taken place in 10/27 responses). Although the analysis was anonymised it should be noted that responses were identifiable when initially returned. Further evaluation at one year will hope to achieve more complete data and this feedback will be performed at the completion of each cycle to give an indication of long-term effectiveness.

To our knowledge, this is the first mentoring initiative to be formally organised by an international subspecialty radiology society. Its strengths are in its attempt to harness the unique qualities of the ESHNR, by bringing together the benefits of international diversity with a common interest and focus on head and neck radiology. Although the context differs to previous experiences of mentoring in radiology practice [9, 10, 14–24], the early feedback indicates that there is a positive experience for mentors and mentees as well as potential benefits for the ESHNR. It could be argued that there is also wider gain to the clinical practice and local institutions of the members involved.

Although there is relatively little on-going demand on resources once the website, documentation and organisational infrastructure are in place, it is appreciated that the core team, mentors and mentees have already saturated working schedules, which limits time for these activities. The cost of programme administration has been incorporated within pre-existing arrangements. The programme is still in an early phase, and it is not possible to determine whether the benefits outweigh the resources involved, which will become evident with further evaluation and feedback following completion of the first-year cycle.

There is currently little evidence base for the positive impact of mentoring schemes in medicine, and the emphasis has previously been on academic medicine. A systematic review found four studies describing the importance of mentorship [28–31] and eight studies reporting an influence on personal development and career enhancement [17, 30, 32–37]. Whilst the potential benefits of mentoring in radiology have been proposed in a number of reviews [9, 10, 14–24], there is relatively little evidence on outcomes. Amongst the limited number of studies that reported outcomes of mentoring programmes, Illes et al. demonstrated positive effects on academic development and patient care in junior academic radiologists [17], whilst mentoring was valued by residents [23, 24] and program directors viewed mentorship as an importance resource [16, 24].

Several potential challenges to the ESHNR mentoring programme have been identified, and these may influence outcomes. Some of these are contextual, such as the international differences in institutional frameworks, accreditation and working environments possibly impeding discussions, although the potential to learn and understand the variation may be seen as an advantage. Similarly, although the programme was generally conducted in the English language, there were language constraints in some cases, which required the pairing of mentors and mentees from the same country. Other challenges are more generic, for instance finding adequate time for the organisation and preparation for mentoring within busy schedules. It may be possible to address this by the recruitment of peri-retirement mentors who will have tremendous experience and more time. In the future, differing approaches to the matching process may also be considered, such as assembling a wider “mentorship team” to give different perspectives or to give the mentees some input [23]. Further mechanisms may also be required to address any mentorship pairings which are found not to be constructive during the course of the programme. A follow-up e mail contact with the mentor and mentee at one month after engagement is now planned in order to detect any difficulties in establishing the mentoring relationship.

## Conclusion

We have documented the process of establishing a mentoring programme for an international subspecialty society, together with an early feedback survey of mentors and mentees. The early experience is encouraging and suggests that it is both useful and sustainable. There is further scope for the dissemination of mentoring resources and mentoring fora, and the prospect of face-to-face mentoring lectures, discussions and social meetings in the post-COVID 19 era is awaited with anticipation. The mentoring programme will attempt to adapt and evolve on the basis of regular feedback, and its success will also be judged on its retention and growth of mentors and mentees. Finally, our strategy may be generalizable to other international subspecialist radiology and other medical societies.

## Data Availability

All data generated or analysed during this study are included in this published article.

## Abbreviations

ESHNR: European Society of Head and Neck Radiology
ESR: European Society of Radiology
SQUIRE-EDU: Standards for Quality Improvement Reporting Excellence in Education
